# Line Orientation Adaptation: Local or Global?

**DOI:** 10.1371/journal.pone.0073307

**Published:** 2013-08-30

**Authors:** Elena Gheorghiu, Jason Bell, Frederick A. A. Kingdom

**Affiliations:** 1 University of Stirling, Department of Psychology, Stirling, Scotland, United Kingdom; 2 Research School of Psychology, Australian National University, Canberra, Australia; 3 School of Psychology, University of Western Australia, Perth, Australia; 4 McGill Vision Research, Department of Ophthalmology, McGill University, Montreal, Canada; University of Sussex, United Kingdom

## Abstract

Prolonged exposure to an oriented line shifts the perceived orientation of a subsequently observed line in the opposite direction, a phenomenon known as the tilt aftereffect (TAE). Here we consider whether the TAE for line stimuli is mediated by a mechanism that integrates the local parts of the line into a single global entity prior to the site of adaptation, or the result of the sum of local TAEs acting separately on the parts of the line. To test between these two alternatives we used the fact the TAE transfers almost completely across luminance contrast polarity [1]. We measured the TAE using adaptor and test lines that (1) either alternated in luminance polarity or were of a single polarity, and (2) either alternated in local orientation or were of a single orientation. We reasoned that if the TAE was agnostic to luminance polarity and was parts-based, we should obtain large TAEs using alternating-polarity adaptors with single-polarity tests. However we found that (i) TAEs using one-alternating-polarity adaptors with all-white tests were relatively small, increased slightly for two-alternating-polarity adaptors, and were largest with all-white or all-black adaptors. (ii) however TAEs were relatively large when the *test* was one-alternating polarity, irrespective of the adaptor type. (iii) The results with orientation closely mirrored those obtained with polarity with the difference that the TAE transfer across orthogonal orientations was weak. Taken together, our results demonstrate that the TAE for lines is mediated by a global shape mechanism that integrates the parts of lines into whole prior to the site of orientation adaptation. The asymmetry in the magnitude of TAE depending on whether the alternating-polarity lines was the adaptor or test can be explained by an imbalance in the population of neurons sensitive to 1^st^-and 2^nd^-order lines, with the 2^nd^-order lines being encoded by a subset of the mechanisms sensitive to 1^st^-order lines.

## Introduction

Visual aftereffects characterize the phenomenon in which the appearance of a stimulus is altered following adaptation to a slightly different stimulus. As an appearance-based psychophysical tool, visual after-effects are useful for studying how visual stimuli are represented in the brain. Visual aftereffects have been used to reveal the nature of the internal representation of low-level features such as orientation [2], [3], [4], [5], [6], and intermediate-to-high level features such as curves [7], [8], shapes [9], [10], [11], [12] and faces [13], [14].

A classical and extensively studied visual after-effect is the tilt after-effect or TAE. With the TAE, prolonged adaptation to an oriented stimulus such as a line or grating causes a shift in the apparent orientation of a subsequently presented stimulus in a direction away from that of the adaptor [1], [2], [3], [4], [5], [6], [15], [16], [17], [18], [19], [20]. As the orientation difference between adaptor and test stimuli is varied, the TAE increases in magnitude for small orientation differences and decreases for larger differences, with no net effect when the adaptor and test orientations are identical. It is generally believed that the TAE is caused by a change in the shape of the response distribution of orientation-selective neurons in primary visual cortex (V1), as a result of either fatigue, gain reduction or lateral inhibition [21], [22], [23], [24], [25], [26].

In this communication we aim to determine the site of the TAE for line stimuli, by considering how the local parts of a line contribute to the line TAE. To understand the rationale behind our experiments, we begin by considering an interesting property of the TAE. Although the TAE has been shown to be selective for chromaticity [27], [28], [29], [30], [31], [32], [33], an early study by Magnussen and Kurtenbach [1] found over 80% transfer of the TAE from black adapting bars to white test bars (1.3 deg length, 0.09 deg width) and vice-versa, and between black-white and white-black edges. This degree of non-selectivity to luminance polarity for simple orientation adaptation is surprising given that it is the higher not lower stages of shape-processing that as a rule are agnostic to luminance polarity. For example, whereas Gheorghiu and Kingdom [34], [35] found that the shape-frequency and shape-amplitude after-effects, which are believed to be mediated by the intermediate-level shape-feature of curvature, are both selective to luminance-contrast polarity, radial-frequency amplitude after-effects [36] and some figural after-effects [37], both of which are arguably mediated by higher-level shape-features, are not [36]. Thus it is possible that the TAE for line stimuli is mediated by a relatively high-level global shape mechanism that integrates the local parts of the line into a single global entity prior to or at the site of adaptation. On the other hand the line TAE might be the result of the sum of local TAEs acting separately on the individual parts of the line. If so, these local TAEs must somehow be integrated to produce the appearance of an unfragmented line shifted in overall orientation, a process explored by Meese and Georgeson [38] in their study of how local, component-grating TAEs are integrated into the global perceived structure of plaids.

**Figure 1:**
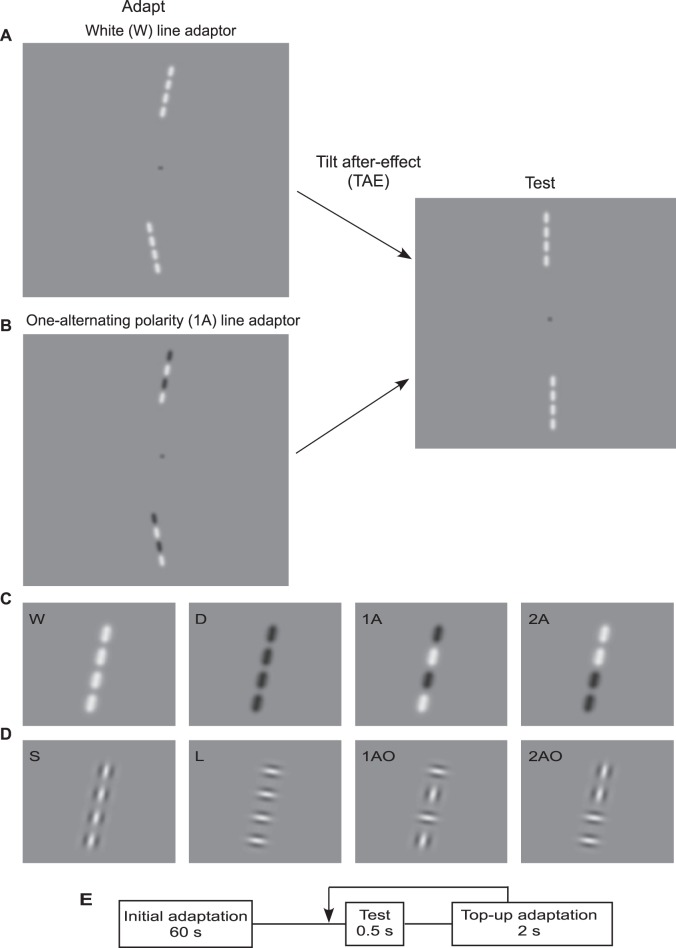
Stimuli used in the experiments. One can experience the tilt after-effect (TAE) obtained with single polarity line adaptors (**A**) and one-alternating polarity line (**B**) by moving one’s eyes back and forth along the markers located midway between the pair of adapting lines (left) for about 60s, and then shifting one’s gaze to the middle of the single single polarity line test (right). (**C**) Example lines made of either all white (W), all dark (D), one-alternating polarity (1A) and two-alternating polarity (2A) micropatterns. (**D**) Example lines in which the Gabor patches were either all tangentially oriented to the line’s path, termed ‘snake’ (S), all orthogonally oriented to the path, termed ‘ladder’ (L), alternating in orientation every other element (1AO) or every two consecutive elements (2AO). (e) Schematic representation of the adapting and test procedure - see text for details.

To test between the global and local TAE alternatives we have measured TAEs using adaptor and test lines that either alternate in luminance polarity along their length or are of a single polarity (see Fig. 1). Our reasoning is that if line TAEs are produced locally and then integrated, it should not matter if the parts of the line adaptor alternate in polarity since polarity is discarded at the level of the TAE, i.e. locally. If it turns out however that an alternating polarity adaptor produce a relatively weak TAE in a single polarity test, then this is evidence against the local TAE explanation and in favour of the global TAE explanation. We have also measured the strength of the TAE using adaptor and test lines that alternate in *orientation* not polarity, to further elucidate the contribution of local versus global TAEs for line stimuli.

## Methods

### Participants

Six observers participated in this study (five in Experiment 1 and 2 and four in Experiment 3): two of the authors (JB and EG) and four observers (AW, CZ, MS and PM) who were naive with regard to the experimental aims. All subjects had normal or corrected-to-normal visual acuity. Observers gave their written informed consent prior to participating in this study and were treated in accordance with the Declaration of Helsinki. The research protocol was approved by the McGill University Health Centre Research Ethics Office and the Australian National University Human Ethics Committee.

### Apparatus and Stimuli

Stimuli were created using Matlab (version 7.6) and loaded into the frame-store of a ViSaGe video-graphics card (Cambridge Research Systems, UK). Stimuli were presented on a calibrated, gamma-corrected Sony Trinitron G400 monitor with a screen resolution of 768×1024 pixels and a refresh rate of 100 Hz. This screen resolution with a viewing distance of 115 cm resulted in each pixel subtending 1 arcmin. The mean luminance of the monitor was 50.4 cd/m^2^.

The TAE was measured using line stimuli constructed from strings of four micropatterns. The adapting and test stimuli consisted of pairs of lines presented 3 deg above and below fixation, on a background of the same mean luminance, as shown in Figures 1A and 1B. The adapting lines subtended 1.84 deg of visual angle and were oriented 10 deg clockwise and 10 deg anticlockwise from vertical.

In Experiment 1 the lines were made of elongated Gaussians micropatterns generated according to a Gaussian function: G(x) = exp [–x^2^/(2σ^2^) ], where x is the distance along the major axis and σ (sigma) the space-constant. The luminance profile of the Gaussian perpendicular to its major axis had a sigma of 0.1 deg. Along its major axis, the upper half of the Gaussian envelope was applied to the outer edge of a circular aperture of radius 0.1 deg centered on the middle of the micropattern. This arrangement served to preserve the luminance micropattern’s orientation anisotropy. The center-to-center spacing between adjacent micro-patterns along the path of the line was 0.5 deg. The luminance contrast of each micropattern was set at 90% (i.e., a Weber contrast of 0.8): +90% for ‘white’ and −90% for ‘dark’. We varied the number of consecutive white and dark micropatterns along the length of the line. Lines were either all white (W), all dark (D), one-alternating polarity (1A) and two-alternating polarity (2A) micropatterns, as shown in Fig. 1C.

In Experiment 2 the lines were made from odd-symmetric (i.e. d.c. balanced) Gabors with a two-dimensional sigma of 0.1 deg and luminance spatial frequency 5 c/deg. As in Experiment 1, contrast was 90%. The center-to-center spacing between adjacent Gabors patches along the line contour was 0.5 deg. We varied the orientation of consecutive Gabors along the path of line contour, with Gabors tangential to the line’s path, termed ‘snake’ (S), orthogonal to it, termed ‘ladder’ (L), or alternating in orientation every other element (1AO) or every two consecutive elements (2AO). Example stimuli are shown in Fig. 1D.

### Procedure

A staircase procedure was employed to measure the TAE. Each session started with an initial adaptation period of 60s, followed by a repeated test of 0.5s duration interspersed with top-up adaptation periods of 2s to reinforce the initial adaptation. A schematic representation of the adapting and test procedure is shown in Fig. 1E. Each test period of 0.5s (signaled by a tone) was preceded and followed by 0.1s blank screen. During the initial and top-up adaptation periods, the spatial location of each set of lines was horizontally jittered (up to +/−30 arcmin, randomly drawn from a rectangular distribution) every 0.5s in order to prevent the formation of afterimages. The spatial location of the test lines was also randomly assigned (again up to +/−30 arcmin) every test period. Example non-static line stimuli are shown in the Supporting Information section for white adaptor/test (Movie S1) and one-alternating polarity adaptor and white test (Movie S2). The location of the fixation cross was not jittered across trials. Subjects were required to fixate on the marker placed between each pair of lines for the entire session. A head and chin rest helped to minimize head movements. On each test trial (signaled by a tone) subjects indicated via a button press which of two lines was more clockwise from vertical. Following the observer’s response, the staircase procedure adjusted the orientation of the upper and lower test lines in a direction opposite to that of the response, i.e. towards the point of subjective equality (or PSE). For the first 5 trials, a step size of 1 deg was added to or subtracted from the orientation of each test line; thereafter 0.5 deg steps were used. The relative orientations of the upper and lower test lines were symmetrical about vertical, with the same angular change added to one and subtracted from the other. The session was terminated after 25 trials. We used a staircase method that was terminated after a fixed number (25) of trials, rather than a fixed number of reversals in order to have the same total amount of adaptation for each condition. On each trial the computer recorded the angular difference between the two lines. The angular difference over the last 20 trials was used to calculate the average angular difference and standard error at the PSE in each condition. For each *with-adaptor* condition we made five measurements. In addition, using the same timing protocol we measured for each condition the mean angular difference at the PSE in the absence of the adapting stimulus (i.e. contrast of the adaptor was set to zero), that is the *no-adaptor or baseline condition*.

To obtain an estimate of the size of TAE we first calculated the difference between each ‘with-adaptor’ angular difference at the PSE and the mean of the ‘no-adaptor’ angular difference at the PSE. We then calculated the mean and standard error of these differences across the five measurements. These standard errors are the ones shown in the graphs.

## Experiment 1: Effect of alternating luminance contrast polarity

Here we examine whether the luminance contrast polarities of the parts of a line are a determinant of the line TAE. We used adaptors that were either all-white (W), all-dark (D), one-alternating (1A) or two-alternating (2A) in polarity, as shown in Fig. 1B. TAEs were measured in test lines that were either all white (W) or one-alternating-polarity (1A).

**Figure 2:**
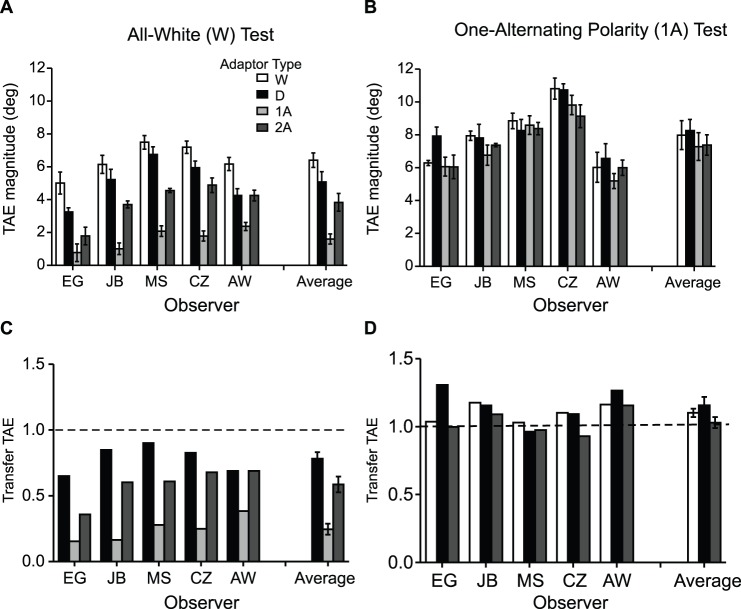
Results for Experiment 1: TAEs induced in all-white test lines (**A**) and one-alternating polarity test lines (**B**), by all-white (white bars), all-dark (black bars), one-alternating (light gray bars) and two-alternating polarity (dark gray bars) line adaptors, for each observer and the average across observers. (**C–D**) Normalized TAE obtained for each ‘different’ adaptor-and-test condition to the after-effect obtained using the ‘same’ adaptor-and-test condition, for each observer and the average across observers. One can think of this measure as the amount of transfer of the after-effect in the ‘different’ condition. Transfer of TAE obtained in the different adaptor-and-test condition normalized to the after-effect obtained with (**C**) all-white adaptor-and-test condition and (**D**) one-alternating polarity adaptor-and test condition. A transfer value of 1 (dashed lines) indicates that the after-effects obtained in the ‘different’ and ‘same’ adaptor/test conditions are similar in magnitude.

Figure 2 shows TAEs induced in all-white (Fig. 2A) and one-alternating polarity (Fig. 2B) test lines, by all-white (white bars), all-dark (black bars), one-alternating (light gray bars) and two-alternating polarity (dark gray bars) line adaptors, for five observers and for the average across observers. The results indicate that TAEs with all-white tests were smallest with one-alternating polarity adaptors, increased slightly for two-alternating polarity adaptors, and were largest for all-white and all-black adaptors (Fig. 2A). On the other hand, TAEs obtained with one-alternating polarity *tests* were similar in magnitude for all adaptor types, and, importantly, large (Fig. 2B).

In order to obtain an overall picture of the difference between the various adaptor-and-test conditions, we normalized the after-effect obtained for each ‘different’ adaptor-and-test condition to that of the associated ‘same’ adaptor-and-test condition, for each observer. One can think of this as the amount of transfer of the after-effect in the ‘different’ condition. For example, for the all-white test condition (Fig. 2C) the TAE transfer was calculated as the aftereffect obtained using a different adaptor normalized to that obtained with the same, i.e. all-white adaptor, for each observer. Fig. 2D shows the transfer for the one-alternating polarity test condition for all observers, with the mean transfer shown on the right. A value of 1 (dashed lines in Fig. 2C and 2D) indicates that the ‘different’ and ‘same’ after-effects had the same magnitude. The results indicate complete transfer of aftereffects across various adaptors in the one-alternating polarity test condition (Fig. 2D) and reduced transfer in the all-white test condition, specifically ∼24.5% with one-alternating polarity adaptors, 58% with two-alternating polarity adaptors and ∼80% with all-dark adaptors (Fig. 2C).

The relatively good transfer of TAEs across different polarities (∼80% black bar in Fig. 2C) replicates Magnussen and Kurtenbach [1] and indicates that the TAE is weak or almost non-selective to luminance contrast polarity. Of principle interest however are the results obtained with one-alternating polarity adaptors and single-polarity tests. This combination produces only weak TAEs (light gray bars in Fig. 2A), which goes against the idea that line TAEs are the sum of its part TAEs. Instead, the results suggest that the local parts of a line are integrated prior to the site of orientation adaptation. We will return to an examination of these results in the Discussion.

## Experiment 2: Effect of alternating local orientation

Here we investigate the role of local orientation alternation for the encoding of global line orientation. Line stimuli comprised Gabor patches that were arranged either collinearly, termed ‘snake’ (S), or orthogonally, termed ‘ladder’ (L), along the line. Lines could also alternate in orientation every element (1AO) or every other element (2AO). Using four types of adaptors: snakes (S), ladders (D), one-alternating- (1AO) and two-alternating-orientation (2AO) we measured TAEs induced in snake test (S) and one-alternating orientation (1AO) test lines.

**Figure 3:**
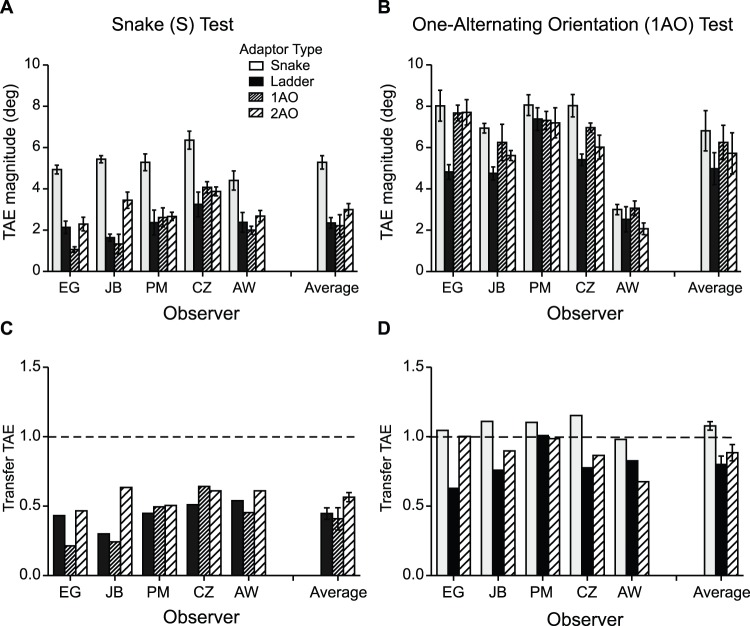
Results for Experiment 2: TAEs induced in snake (**A**) and one-alternating-orientation test lines (**B**) by snake (white), ladder (black), one-alternating (dense hatched) and two-alternating-orientation (sparse hatched) adaptors, for each observer and the average across observers. (**C–D**) Transfer of TAE obtained in the different adaptor-and-test condition normalized to the after-effect obtained with (**C**) snake adaptor-and-test condition and (**D**) one-alternating polarity adaptor-and test condition, for each observer and the average across observers. A transfer value of 1 (dashed lines) indicates that the after-effects obtained in the ‘different’ and ‘same’ adaptor/test conditions are similar in magnitude.

Figure 3 shows TAEs obtained with snake (Fig. 3A) and one-alternating-orientation test lines (Fig. 3B), using snake (white), ladder (black), one-alternating (dense hatched) and two-alternating-orientation (sparse hatched) adaptors, for five observers and the average across observers. Again, we normalized the results to the ‘same’ adaptor-and-test condition for each observer and these are shown in Fig. 3C for snake and Fig. 3D for one-alternating orientation test lines.

First consider the orientation analog of the opposite polarity adaptor-test condition in Experiment 1. Unlike the ∼80% transfer we found across opposite polarities, transfer from ladder adaptors to snake test contours was relatively weak at ∼44%. Apart from this difference, the results with orientation closely mirror those obtained with polarity. As with polarity, TAEs are relatively small for alternating-orientation adaptors combined with single-orientation snake tests, but not the other way round (compare dense hatched bars in Fig. 3A with white bars in Fig. 3B). In fact there is almost complete transfer of the TAE across all types of adaptor when combined with one-alternating-orientation tests (average transfer ∼1.08% for snake, ∼80% for ladder and ∼89% for two-alternating-orientation adaptors - see Fig. 3D). As with polarity, the asymmetry in the magnitude of TAEs depending on whether the alternating orientation line is an adaptor or a test goes against the idea that line TAEs are the sum of its part TAEs. Instead, the results suggest that the local parts of a line are integrated prior to the site of orientation adaptation.

## Experiment 3: Effect of positional jitter on global line processing

Both previous experiments go against the idea that the parts of the lines adapt separately to orientation, and favour instead the idea that the parts are integrated into lines prior to adaptation. If the parts of the adaptor are normally integrated prior to adaptation, it follows that if they are spatially separated during adaptation, the resulting TAEs should be reduced. Here we test this prediction.

**Figure 4:**
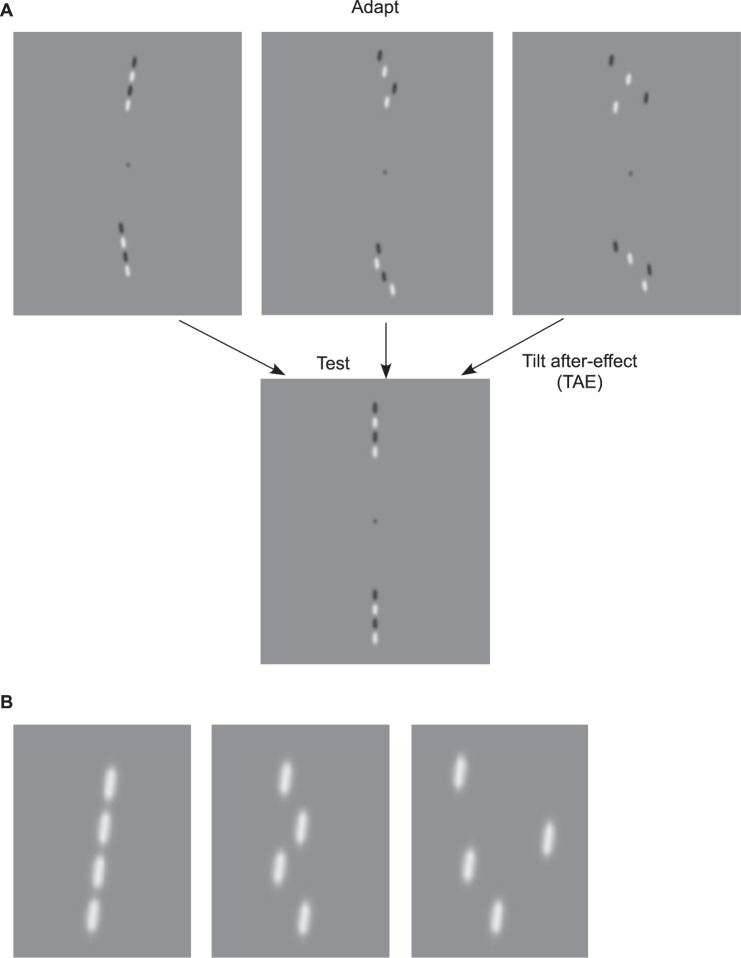
Example parts-jittered lines used in Experiment 3. One can experience the TAE obtained with (**A**) jittered one-alternating polarity line adaptor for large (right), small (center) and no (left) jitter condition and no jittered one-alternating polarity line test. (**B**) Example parts-jittered white lines for large (right), small (center) and no (left) jitter condition.

We used adaptors in which the positions of the micropatterns were individually jittered alternately to opposite sides (i.e. to the left ‘−‘ and right ‘+’) of the nominal line, by a random amount within a specified range. This is the ‘parts-jittered’ condition. The control condition was a whole line jittered by the same amount, termed the ‘whole-jittered’ condition. We used three values of jitter: no jitter, +/−25 arcmin (small) and +/−50 arcmin (large) jitter. Example parts-jittered lines are shown in Fig. 4. There were four adaptor and test conditions: two for the polarity experiment (i.e. one-alternating polarity and white line - see Fig. 4) and two for the orientation experiment (i.e. snake and one-alternating orientation lines). In all conditions, the adaptor and test lines were of the same type. The test lines in all conditions were whole lines (i.e. no local jitter of micropatterns) that were jittered by the same amount as the adapting lines (Fig. 4A).

**Figure 5:**
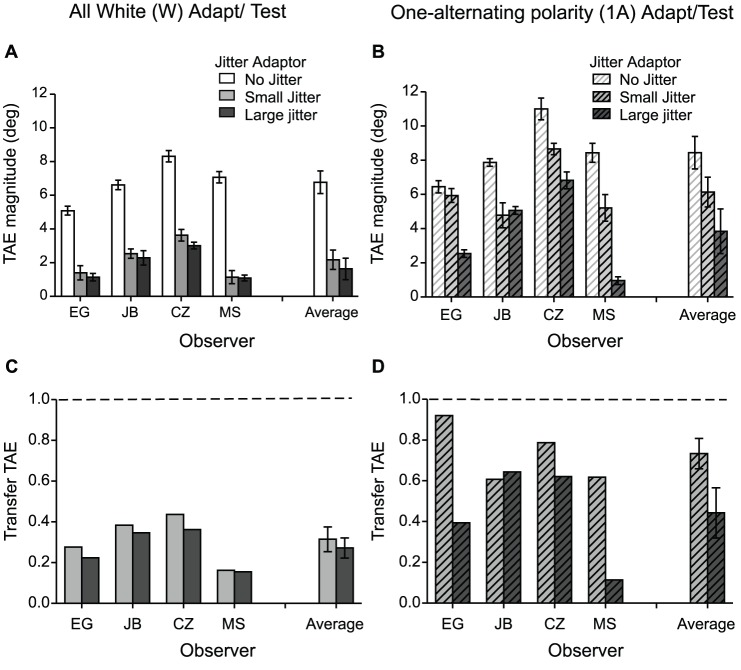
Results for Experiment 3– polarity condition. TAEs obtained with (**A**) all white lines adaptor-and-test for small (light gray bars), large (dark gray bars) and no jitter (white bars) adaptor conditions, for each observer and the average across observers. (**B**) one-alternating polarity adaptor-and-test lines for small (hatched light gray bars), large (hatched dark gray bars) and no jitter (hatched white bars) adaptor conditions, for each observer and the average across observers. (**C–D**) The normalized results and average transfer across observers for (**C**) all white and (**D**) one-alternating polarity lines, respectively.

**Figure 6:**
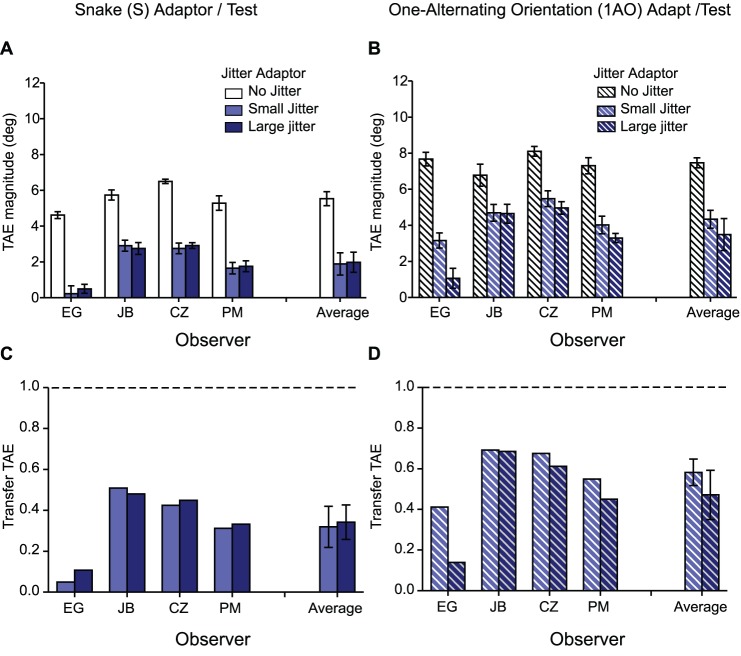
Results for Experiment 3– orientation condition. TAEs obtained with (**A**) snake adaptor and test for small (light blue bars), large (dark blue bars) and no jitter (white bars) adaptor conditions, for each observer and the average across observers. (**B**) one-alternating orientation adaptor and test lines for small (hatched light blue bars), large (hatched dark blue bars) and no jitter (hatched white bars) adaptor conditions, for each observer and the average across observers. (**C–D**) The normalized results and average transfer across observers for (**C**) snake and (**D**) one-alternating orientation lines, respectively.

Figure 5 shows TAEs obtained with all white (Fig. 5A) and one-alternating polarity lines (Fig. 5B) for small (light gray bars), large (dark gray bars) and no jitter (white bars) adaptor conditions, for four observers and for the average across observers. The normalized results and average transfer across observers are shown in Fig. 5C and 5D, respectively. Figure 6 shows the corresponding TAEs obtained with snake (Fig. 6A) and one-alternating orientation lines (Fig. 6B), with the normalized results in Figures 6C and 6D. Clearly, spatially jittering the adaptor micropatterns significantly reduces the TAEs, especially for the larger jitter range (compare dark and light gray bars in Fig. 5C; also compare hatched dark and light gray bars in 5D). For white lines the TAEs are reduced to an average of ∼27% of the whole-line condition (dark gray bar in Fig. 5C), while for the one-alternating polarity lines, the reduction is on average ∼44% (hatched dark gray bar in Fig. 5D). With the orientation conditions TAEs are reduced to an average of ∼33% for the snake (dark blue bar in Fig. 6C), and ∼47% for alternating-orientation lines (hatched dark blue bar in Fig. 6D).

We will discuss these results further in the next section but in summary the results of the jitter experiment add further support to the idea that the parts of lines are integrated prior to orientation adaptation.

## Discussion

Using lines constructed from elongated Gaussian elements we tested whether the tilt aftereffect (TAEs) resulted from adaptation to the whole line or separately to its parts. We found that whereas line adaptors showed strong TAEs even when adaptor and test were of opposite luminance polarity (confirming the previous report from [1]), adaptors consisting of alternating polarity elements produced relatively weak TAEs in single-polarity (all-white) tests. This is inconsistent with the idea that line TAEs are the sum of its part TAEs, since if TAEs are largely agnostic to polarity and are the sum of its parts, it should not matter that polarity alternates between the parts. Rather, the results suggest that the parts of the lines are integrated into wholes prior to orientation adaptation. This conclusion receives additional support from the finding that when the adaptor parts were distributed across space while preserving their orientations, TAEs in line tests were significantly reduced.

Our one-alternating polarity adaptors did however produce large TAEs in the same, i.e. one-alternating polarity tests. The most parsimonious explanation for this finding is that the TAEs in this condition are mediated by line-sensitive mechanisms that are agnostic to local luminance polarity, but which nevertheless integrate the parts of the line into a whole, similar to mechanisms sensitive to single-polarity lines. This explanation leads to a consideration of another important property of the data: the asymmetry in the magnitude of the TAEs between alternating-polarity adaptors combined with all-white tests (which produce small TAEs – see Fig. 2A light grey bars), and all-white adaptors combined with alternating-polarity tests (which produce large TAEs – see Fig. 2B white bars). The asymmetry cannot be explained by weak adaptation to dark elements, since an all-dark adaptor induces large TAEs in an all-white test (∼80% transfer – see Fig. 2C dark bars). So what causes it? One possibility is that there is an imbalance in the populations of neurons sensitive to the two classes of line, with one population sensitive exclusively to single-polarity lines and the other sensitive to both single-polarity and alternating-polarity lines. Thus an all-white adaptor will stimulate both populations, resulting in a large TAE not just for an all-white test but also for an alternating-polarity line. However, an alternating-polarity adaptor will only stimulate one of the two populations, so not all the neurons sensitive to an all-white test will become adapted, resulting in a relatively small TAE. A corollary to this scheme is that the results suggest that are no neurons sensitive exclusively to alternating-polarity lines: the TAE obtained using an all-white adaptor with a one-alternating polarity test was similar to that obtained using a one-alternating polarity adaptor *and* test (compare white bar with the dashed line in Fig. 2D). In other words if there were neurons exclusively sensitive to alternating-polarity lines, the one-alternating polarity test lines would be detected by at least some unadapted line detectors, which would reduce the size of the TAE.

**Figure 7:**
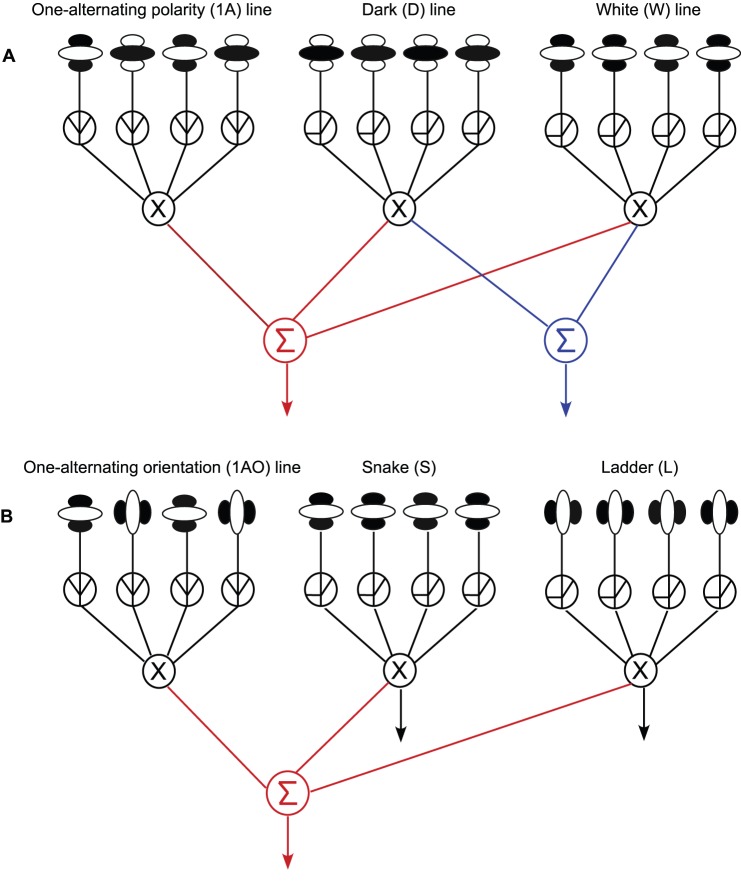
Schematic representation of a neural model the might explain the asymmetry in the TAE obtained in the polarity (A) and orientation (B) conditions, as applied to lines of 4 microelements. The lines are all detected by 2^nd^-order mechanisms that detect changes across space and/or time of the response energies of linear simple-cell-like filters (termed ‘1^st^-order’) that pick up the local luminance/orientation detail in the image. The rectified 1^st^-order signals are combined by the 2^nd^-order filter via AND-gating operation (denoted by X). (**A**) The all-white and all-dark 2^nd^-order responses are combined into a common pathway (denoted by blue). A second pathway is sensitive to all-white, all-black and alternating-polarity lines (denoted by red). The arrangement of pathways explains the asymmetry in the transfer of TAE between alternating-polarity adaptors combined with all-white tests, and all-white adaptors combined with alternating-polarity tests. The scheme for coding lines made from different orientations (**B**), is somewhat different from the scheme for coding lines with different luminance polarities in that snakes and ladders are processed by different pathways (see text for details).

A proposed scheme for coding lines made from different luminance polarities is illustrated in Fig. 7A, as applied to lines of 4 microelements. In the scheme, the lines are all detected by mechanisms that may be termed ‘2^nd^-order’. These 2^nd^-order mechanisms detect changes across space and/or time of the response energies of linear simple-cell-like filters (termed ‘1^st^-order’) that respond to the local luminance detail in the image. Second-order mechanisms are typically modeled in the form of a Filter-Rectify-Filter cascade, in which the 1^st^-order responses are subject to a nonlinearity, such as squaring or rectification, then pooled by a larger-in-scale filter tuned to the orientation and scale of the changes in response-energy – for a recent review see Graham [39]. In the texture domain, 2^nd^-order mechanisms sensitive to modulations of contrast have been found to be sensitive to local luminance polarity, while those sensitive to modulations of local orientation have been found to be agnostic to local luminance polarity [40], [41]. In Fig. 7A, both polarity-sensitive and polarity-insensitive 2^nd^-order mechanisms encode line orientation, via half-wave and full-wave rectification of the 1^st^-order inputs respectively. The rectified 1^st^-order signals are combined by the 2^nd^-order filter via AND-gating or an equivalent operation, consistent with evidence from Gheorghiu and Kingdom [42] for curvature detectors. In Fig. 7A, the all-white and all-dark 2^nd^-order responses are finally combined into a common pathway (denoted by blue), hence the strong transfer of TAEs between dark line adaptors and white line tests (black bars in Fig. 2C). A second pathway is sensitive to all-white, all-black and alternating-polarity lines (denoted by red). The arrangement of pathways explains the asymmetry in the transfer of TAE between alternating-polarity adaptors combined with all-white tests (light grey bars in Fig. 2C), and all-white adaptors combined with alternating-polarity tests (white bars in Fig. 2C).

The detrimental effect on the TAE of positionally jittering micropattrerns (Experiment 3) brings further support to the idea that the parts of our lines are integrated prior to orientation adaptation. Why? Remember that the whole-line comparison adaptor was jittered over the same overall spatial extent as the separately jittered parts adaptor, so a linear line-sensitive mechanism would be stimulated equally by both types of stimulus during adaptation. The detrimental effect of jittering the parts therefore suggests that the parts are combined via a nonlinearity such as AND-gating, which for lines is a form of collinear facilitation. In other words a continuous line is ‘greater than the sum of its parts’, and so adapting to its separated parts will produce smaller TAEs in line tests than adapting to the continuous line. Neurophysiological evidence supports the existence of such an AND-gating nonlinearity. In some V1 neurons, oriented lines placed outside of the classical receptive-field enhance its response, for example when the orientations are collinear with the preferred classical receptive-field orientation [43], [44] or when orthogonally-oriented to the preferred classical receptive-field orientation, termed cross-orientation facilitation [45], [46]. These contextual interactions depend not only on the relative orientations of the elements in the surround and classical receptive-field but also on their relative spatial positions [47]. The finding that the TAEs for jittered one-alternating lines (Fig. 5D and 6D) were about twice that obtained with single-polarity lines (Fig. 5C and 6C) shows that there is a larger tolerance for jitter in the former; this might be due either to larger polarity-agnostic line receptive-fields or a ‘softer’ integrating non-linearity.

In addition, neurophysiological studies that measured receptive-field sizes of orientation-selective V1 neurons in macaque as a function of eccentricity (i.e. the distance between the receptive-field center and fovea) have also shown that for eccentricities smaller than ∼6 deg the receptive-field sizes of orientation-selective V1 neurons are typically small, between 0.5–1.5 deg [48], [49], [50]. For orientation-selective V2 and V4 neurons the receptive-field sized of V2 neurons were found to be between 0.5–3.5 deg for eccentricities smaller than ∼ 4 deg [48], [50] and between 1–4 deg for V4 neurons for eccentricities smaller than ∼3 deg [49], [50]. Thus, line stimuli (presented at 3 deg eccentricity) made of micropatterns that are spatially jittered for more that 0.5–1.5 deg will not optimally stimulate the small size receptive fields of orientation-selective neurons. In our Experiment 3, both single-polarity/orientation and one-alternating polarity/orientation adapting line stimuli made of micropatterns spatially jittered over a 50 and 100 arcmin range (i.e. +/−25 and +/−50 arcmin respectively) resulted in prominently reduced TAEs. In addition, single-polarity/orientation lines showed a stronger selectivity for the small positional jitter (+/−25 arcmin) than did one-alternating polarity/orientation line stimuli, suggesting that the receptive field sizes of the one-alternating line mechanisms are larger than those of single-polarity/orientation line mechanisms. Indeed, doubling the amount of positional jitter (+/−50 arcmin) for one-alternating polarity/orientation lines resulted in reduced after-effects that were comparable in magnitude with those obtained with same-polarity/orientation line stimuli jittered over a small positional range.

Our proposed scheme applied to lines made of 4 microelements (Fig. 7A) could equally apply to lines with other numbers of elements, both smaller and larger, as TAEs are probably mediated by filters tuned to various line lengths. Moreover, Fig. 7A is not meant to imply that the TAE is tightly tuned to line length per se. Indeed one reason why the two-alternating polarity adaptors produce larger TAEs than the one-alternating polarity adaptors in single-polarity tests (compare dark and light gray bars in Fig. 2A) might be the greater degree of overlap in the range of line-sensitive mechanisms between two- and four-element lines compared to one- and four-element lines.

In Experiment 2 we examined the strength of the TAE using adaptor and test lines that alternated in orientation rather than polarity. As with the polarity results in Experiment 1, we found a similar asymmetry in the magnitude of the TAEs depending on whether the alternating orientation line was an adaptor or a test. Once again, this asymmetry suggests that the local parts of a line are integrated prior to the site of orientation adaptation. The one difference between the results obtained with orientation (Experiment 2) and polarity (Experiment 1) was the TAE transfer across opposite orientations, which was found to be ∼44%, compared to ∼80% for opposite polarities (compare black bars in Fig. 2C and 3C). The reduced TAE transfer between ladder adaptors and snake tests suggests that snake and ladder lines are not processed by the same mechanism. Thus our proposed scheme for coding lines made from different orientations, shown in Fig. 7B, is somewhat different from the scheme for coding lines with different luminance polarities in that snakes and ladders are processed by different pathways (denoted by separate black arrows in Fig. 7B). Similar to the polarity scheme, Fig. 7B shows that snakes, ladders and alternating-orientation lines are all processed by a single common pathway (denoted by red).

The finding that TAEs obtained with one-alternating orientation adaptors and snake tests (see Fig. 3A dense hatched bars) are prominently reduced is in keeping with previous findings from another after-effect, the shape-frequency after-effect, in which adaptation to a sine-wave-shaped contour causes a repulsive shift in the perceived shape-frequency of a test contour with a slightly different shape frequency [51]. Gheorghiu and Kingdom [51] found that interleaving opposite orientations into a single curved contour adaptor disrupted the shape-frequency aftereffect more than when simply removing half the orientations.

Do other visual aftereffects manifest an asymmetry in the transfer between different types of cues? Asymmetric transfer of after-effects has been found for the dynamic motion after-effect between luminance modulation (1^st^-order) and contrast modulation (2^nd^-order) and among different types of 2^nd^-order cues [52]. Asymmetric transfer of after-effects has also been found between real and illusory contours for both the tilt [5] and curvature [53] after-effects. The illusory contour studies found that real contour adaptors produced after-effects in illusory contour tests that were as strong as, or even stronger than those produced by illusory contour adaptors, while illusory contour adaptors produced weaker after-effects in real contour tests compared to real contour adaptors. The authors of both studies argued for a similar explanation for the asymmetry as advanced here. Additional support for this explanation comes from neurophysiological studies showing that neurons in area V2 that respond to illusory contours constitute approximately 40% of the neural population that respond to real contours [54], [55].

On the other hand, Georgeson and Schofield [56] found almost complete, symmetric transfer of both the TAE and the contrast-reduction after-effect between luminance modulated (LM) and contrast modulated (CM) gratings, leading them to suggest that LM and CM information is combined at the site of adaptation. However, when LM and CM gratings were combined in-phase or out-of-phase these authors found no evidence for cancellation, nor for 'phase-blindness' and hence no evidence that information about LM and CM is pooled. Thus, these authors conclude that although LM and CM signals are carried by separate channels, they share a common adaptation mechanism that accounts for the almost complete transfer of TAE.

Our results demonstrate that the TAE for line stimuli is mediated by a relatively high-level global shape mechanism that integrates the local parts of the line into a single global entity prior to the site of adaptation. Brain imaging studies that measure orientation-selective adaptation with fMRI, and which allow one to make inferences about the neural activity at the subpopulation level, have not reached a consensus as to which visual areas are involved in orientation-specific adaptation. While some studies report that orientation-specific adaptation of the fMRI response largely occurs in primary visual cortex (V1) [57], [58] others have shown no orientation-specific adaptation effects in V1 but instead increasing adaptation effects along the hierarchy of extra-striate visual areas (V2, V3, V4V, VO1, LO1) [59], [60]. Larson et al. [60] showed that while for 1^st^-order (luminance) gratings stimuli, the adaptation was no larger in extra-striate areas than in V1 thus implying that orientation-selective 1^st^-order adaptation originates in V1, for 2^nd^-order stimuli (e.g. contrast modulated) the strength of adaptation was significantly larger in areas VO1, V3A/B and LO1 than in V1 suggesting that 2^nd^-order stimulus orientation was extracted in higher visual areas.

## Supporting Information

Movie S1 **Example TAE obtained with non-static, white adaptor/test.**
(MOV)

Movie S2 **Example TAE obtained with non-static, one alternating polarity adaptor and white test.**
(MOV)
